# Recent advances in the treatment of systemic sclerosis associated interstitial lung disease

**DOI:** 10.3389/fmed.2023.1155771

**Published:** 2023-03-24

**Authors:** Antoniya Kamenova, Argyris Tzouvelekis, George A. Margaritopoulos

**Affiliations:** ^1^Interstitial Lung Disease Unit, London North West University Hospital HT, London, United Kingdom; ^2^Respiratory Medicine Department, University of Patras, Patras, Greece

**Keywords:** interstitial lung disease, systemic sclerosis, cyclophosphamide, rituximab, mycophenolate mofetil, tocilizumab, nintedanib, pirfenidone

## Abstract

Connective tissue diseases (CTDs) are a heterogenous group of systemic inflammatory disorders. The development of connective tissue disease-associated interstitial lung disease (CTD-ILD) is a key complication associated with significant morbidity and mortality. The aim of this review is to explore the pathogenesis of CTD-ILD and summarize the recent evidence from clinical trials for novel treatment options, including the role of antifibrotics and immunomodulatory therapies with a focus on systemic sclerosis associated ILD. Further clinical trials are ongoing to explore combination therapies and more targeted therapeutic options. Clinicians remain faced with the difficult challenge of appropriately selecting patients who will benefit from the available therapies and timing the start of therapy at the most suitable part of the disease course.

## 1. Introduction

Connective-tissue diseases (CTDs) are systemic conditions which are either caused by a specific genetic abnormality causing a defect in connective tissues or by an autoimmune reaction toward connective tissues, leading to symptoms affecting the skin, bone, cartilage, and blood vessels. The high amount of collagen and blood vessels in the lung make it a particularly susceptible target of autoimmune CTDs. Any component of the lung can be involved including airways, blood vessels, lung parenchyma, pleura or respiratory muscles (1). The development of interstitial lung disease (ILD) in autoimmune CTDs is a key complication, associated with significant morbidity and mortality (2). The types of autoimmune CTDs that are associated with the development of ILD are systemic sclerosis, rheumatoid arthritis, idiopathic inflammatory myositis (polymyositis-dermatomyositis and anti-synthetase syndrome), primary Sjogren syndrome, mixed connective tissue disease, and systemic lupus erythematosus, with various frequency (Table 1) (2). Gaining a clearer understanding of the pathogenesis of CTD-ILD paves the way for the development of targeted therapies and their timely application during the pathogenetic process. This review will focus on the pathogenesis of CTD-ILD, particularly SSc-ILD which is more commonly associated with ILD than other CTDs (3), and summarize the recent evidence from clinical trials.

**Table 1:** Autoimmune CTDs and frequency of associated ILD.

Connective tissue disease	Frequency of associated ILD (2)
Systemic sclerosis	+++
Polymyositis/dermatomyositis	+++
Rheumatoid arthritis	++
Primary Sjogren’s disease	++
Systemic lupus erythematosus	++

## 2. Epidemiology of SSc-ILD

The prevalence of systemic sclerosis (SSc) is 50 to 300/million (4, 5) and SSc-ILD develops in more than 50% of cases. The types of SSc are limited cutaneous and diffuse cutaneous based on the degree of skin involvement. In limited cutaneous SSc (lcSSc) there is no skin involvement proximal to the elbows and the knees (with the exception of the face and neck areas that can be involved), while diffuse cutaneous SSc (dcSSc) affects the skin in proximal areas such as the trunk. Both lcSSc and dcSSc can be associated with ILD. ILD develops more frequently in dcSSc patients, but as lcSSc is the more common form of SSc, overall the prevalence of SSc-ILD is similar across both cohorts. The most common pattern of ILD associated with SSc is non-specific interstitial pneumonia (NSIP) (3). Another potential way to stratify the risk of developing SSc-ILD is the autoantibody profile. The risk of developing SSc-ILD is much higher with the presence of anti-topoisomerase antibodies (anti-Scl-70 autoantibodies) as opposed to anti-centromere antibodies which are more related with the development of pulmonary hypertension (5).

## 3. Pathogenesis – The transition from inflammation to fibrosis

The pathogenesis of CTD-ILD remains incompletely understood but is known to be a complex interplay between environmental and genetic factors. The first stage of the pathogenesis of CTD-ILD is inflammation and alveolar epithelial damage, which may be triggered by infection, by inhalation of irritating agents or by gastro-esophageal reflux disease in genetically susceptible individuals (3).

One of the major genes that have been implicated in the pathogenesis of idiopathic pulmonary fibrosis, *MUC5B*, has also been shown to play a role in the pathogenesis of CTD-ILD, particularly RA-ILD (6). The *MUC5B* promoter variant RS35705950, which has been found to be associated with RA-ILD (7), drives overexpression of MUC5B protein which affects the cilia clearance mechanisms of the lung (8). Mutations leading to telomere shortening have been found to be related to different types of pulmonary fibrosis, including CTD-ILD (9). Shorter leukocyte telomere length has been found to correlate with more rapid decline in lung function and shorter duration of transplant-free survival in patients with interstitial pneumonia with autoimmune features (IPAF) and CTD-ILD (10–12). One study identified that a polymorphism in the promoter of the connective-tissue growth factor gene is significantly more common in patients with systemic sclerosis suggesting a potential pathogenetic mechanism (13). Further genetic studies have implicated CD226 and IRF5 (14, 15). Genetic studies in different populations have identified several human leukocyte antigens (HLAs) that are associated with systemic sclerosis (16–20).

In these genetically susceptible individuals certain environmental factors trigger the pathogenetic process. These factors include gastro-esophageal reflux disease, infections, noxious chemicals and certain drugs (1). Gastro-esophageal reflux disease is associated with micro-aspirations that lead to repetitive lung injury (21). The noxious chemicals present in tobacco smoke are also known to cause inflammation and associated lung injury (22, 23), but its role in CTD-ILD is not clearly established. Among different types of CTD-ILD, smoking is most strongly associated with RA-ILD (24–26), but studies have also shown that patients with SSc who smoke heavily have increased prevalence of emphysema and decreased survival (27–29). Drugs are also implicated in the development of CTD-ILD (30). This can be challenging for the treating clinician particularly since anti-rheumatic medication such as penicillamine (31), gold (32, 33), sulfasalazine (34), and tacrolimus (35, 36) used to treat a particular type of CTD may lead to the development ILD. It is important to note, however, that particularly in the case of methotrexate, a growing body of evidence is accumulating to exonerate methotrexate in the pathogenesis of ILD (37). In fact methotrexate may protect from the development of ILD, as suggested by retrospective studies (38).

An environmental factor in a genetically susceptible individual acts to trigger the initial process of inflammation. This initial process of potentially reversible alveolitis eventually transforms into irreversible fibrosis. Therefore, it is key to identify markers of active alveolar inflammation that would identify reversible stages of the disease process (3). Surfactant Protein D (SP-D) and Krebs von den Lungen-6 (KL-6) are glycoproteins secreted by type II pneumocytes that can be used as markers of disease progression and also to signify the extent of lung involvement in scleroderma (39). This inflammatory process *via* the activation of various types of immune cells and the triggering of repair mechanisms eventually leads to the increased deposition of extra-cellular matrix, i.e., the development of fibrosis. It is important to note that in the early stages of the disease, inflammatory and pro-fibrotic processes may be occurring alongside each other, which has implications for the choice of therapeutic agents.

A key cell facilitating the increased turnover and eventual deposition of extra-cellular matrix is the resident interstitial pulmonary fibroblast. Interstitial pulmonary fibroblasts get activated *via* multiple pathways, including the transforming growth factor beta (TGF-β) pathway and recruit circulating fibroblasts and myofibroblasts. The crucial role played by the TGF-β pathway is demonstrated by a mouse model in which the TGF-β pathway is suppressed *via* the deletion of the high-affinity type II TGFβ receptor in resident fibroblasts. That mutation led to the complete absence of fibrosis after intrathecal administration of bleomycin (40).

Both innate and adaptive immunity play a role in the pathogenesis of CTD-ILD. A Th2-driven immune response has recently been elucidated, with the proinflammatory cytokines interleukin-4 (IL-4) and interleukin-13 (IL-13) playing a role in the pathogenesis (41). The active humoral response in CTD-ILD can also be illustrated by the multiple antibodies in the sera of patients with CTD-ILD. Different types of autoantibodies could be a marker for the different types of severity of lung fibrosis; anti-topoisomerase antibodies, anti-U11/U12 ribonucleoprotein (RNP) antibodies and anti-Th/To RNP antibodies are associated with an increased likelihood of developing clinically significant fibrosis (42, 43). It is not clear what the underlying pathophysiological mechanism is behind this association, but it is possible that autoantibodies can be used to risk stratify patients and may in the future prove to be suitable therapeutic targets (3). Another important role played by autoantibodies in the pathogenesis of systemic sclerosis is the link between autoantibody-mediated activation of the endothelium and vasculopathy. There are various functional autoantibodies in patients with systemic sclerosis secreted by dysregulated B cells that target endothelial cells, intercellular adhesion molecule 1 (ICAM-1), endothelin type A receptor (ETAR), angiotensin II type I receptor (AT1R) and platelet-derived growth factor receptor (PDGFR). This results in perturbation of the endothelial cells which in turn causes their activation and accelerated apoptosis. The activated endothelial cells release a host of pro-inflammatory cytokines that stimulate the transformation of myofiobroblasts into fibroblasts leading to increased collagen deposition and subsequent tissue fibrosis (44).

## 4. Treatment

While immunosuppressant therapeutic options are well-established for the treatment of the systemic features of autoimmune CTDs, developing therapies that address the ILD manifestation of CTD, which is often the most severe component of the disease, is currently an area of active research. In addition to the considerable evidence base for the use of cyclophosphamide (45) and mycophenolate mofetil (46) in SSc-ILD, major breakthroughs have occurred in the last few years with new evidence accumulating from randomized controlled trials for the benefit of nintedanib (47, 48), rituximab (49), and tocilizumab (50) in SSc-ILD. The majority of clinical trials in CTD-ILD focused on SSc-ILD.

We will explore the findings of recent clinical trials for pharmacological options in CTD-ILD, summarized in Table 2.

**Table 2:** Summary of recent clinical trials for pharmacological therapies in CTD-ILD.

Trial	Year of completion	Study size	Study Design	Condition	Intervention	Primary Outcome	Result	References
UK Trial	2006	*N* = 45	RCT	SSc-ILD	Oral prednisolone, intravenous cyclophosphamide and oral azathioprine versus placebo	Decline in FVC and DLCO at 12 months	There is no statistically significant difference between combination therapy with oral prednisolone, intravenous cyclophosphamide and oral azathioprine versus placebo, but there was a trend toward lung function response with active therapy in SSc-ILD patients	(51)
SLS-I	2006	*N* = 158	RCT	SSc-ILD	Oral cyclophosphamide versus placebo	Decline in FVC at 12 months	There was a statistically significant reduction in FVC decline and in functional outcomes in SSc-ILD patients treated with oral cyclophosphamide, but the difference was not maintained at 24 months, apart from continuously improved dyspnoea	(45)
SLS-II	2016	*N* = 142	RCT	SSc-ILD	Oral cyclophosphamide versus MMF	Decline in FVC at 24 months	There was improvement in FVC with both cyclophosphamide and MMF and the drop-out rate was higher with cyclophosphamide. The first trial to show a benefit of MMF in SSc-ILD.	(46)
SENSCIS	2019	*N* = 576	RCT	SSc-ILD	Nintedanib versus placebo	Decline in FVC at 52 weeks	Nintedanib reduced the rate of decline of FVC compared to placebo in SSc-ILD.	(47)
INBUILD	2019	*N* = 170	RCT	progressing fibrosing autoimmune-ILD	Nintedanib versus placebo	Decline in FVC at 12 months	Nintedanib significantly slowed down the rate of FVC decline in progressing fibrosing autoimmune-ILD and this was maintained beyond 52 weeks. The effect was even more pronounced in patients with UIP-like ILD.	(48)
faSScinate	2016	*N* = 87	RCT	Progressive systemic sclerosis	Tocilizumab versus placebo	Decline in FVC at 48 weeks	Tocilizumab slowed FVC decline in patients with SSc-ILD, but the change was not statistically significant	(52)
focuSSced	2020	*N* = 210	RCT	dcSSc	Tocilizumab versus placebo	Change of mRRS from baseline to week 48	Failed to meet primary end point. Met secondary end point showing statistically significant reduction of the rate of decline of FVC in both the overall population and in the subgroup of patients with SSc-ILD.	(50)
STRATUS	2021	*N* = 40	RCT	SSc-ILD patients receiving stable MMF	Abituzumab versus placebo	Decline in FVC at 12 months	Trial terminated early due to small sample size	(53)
Sircar et al.	2018	*N* = 60	RCT	dcSSc	Rituximab versus cyclophosphamide	Change in FVC at 6 months	Statistically significant improvement in FVC at 6 months in the rituximab arm of the trial compared to decline in FVC in the cyclophosphamide arm of the trial. Serious adverse effects more common with cyclophosphamide.	(54)
RECITAL	2022	*N* = 101	RCT	CTD-ILD	Rituximab versus cyclophosphamide	Decline in FVC at 24 weeks	Reduced rate of decline of FVC at 24 weeks in both arms of the trial, but no statistically significant difference between rituximab and cyclophosphamide. Reduced rate of decline of FVC maintained at 48 weeks for both cyclophosphamide and rituximab.	(49)
SLS-III	2022 (expected)	*N* = 150	RCT	SSc-ILD	Pirfenidone and MMF versus placebo and MMF	Decline in FVC at 18 months	Results awaited	(55)
Wang et al.	2022	*N* = 111	RCT	CTD-ILD	Pirfenidone and immunosuppressants vs. placebo and immunosuppressants	Change in FVC at 24 weeks	Improvement in FVC at 24 weeks in patients with SSc-ILD and inflammatory myopathy-ILD	(56)

SLS-I, Scleroderma Lung Study I; SLS-II, Scleroderma Lung Study II; SLS-III, Scleroderma Lung Study III; SENSCIS, Safety and Efficacy of Nintedanib in Systemic Sclerosis; INBUILD, Efficacy and Safety of Nintedanib in Patients with Progressive Fibrosing Interstitial Lung Disease; APRIL, Abatacept in RA-ILD; faSScinate, A Study of RoActemra/Actemra (Tocilizumab) Versus Placebo in Patients With Systemic Sclerosis; focuSSced, A Study of the Efficacy and Safety of Tocilizumab in Participants with Systemic Sclerosis; STRATUS, Systemic Sclerosis Abituzumab Study; RECITAL, Rituximab versus cyclophosphamide for the treatment of connective tissue disease-associated interstitial lung disease; FVC, forced vital capacity; DLCO, diffusing capacity for carbon monoxide; MMF, mycophenolate mofetil; dcSSc, diffuse cutaneous scleroderma.

Cyclophosphamide is a cytotoxic alkylating agent that is used in the treatment of certain oncological and autoimmune conditions (57). Retrospective studies indicated cyclophosphamide may be beneficial in the treatment of scleroderma-related ILD (SSc-ILD) (58, 59), but there was a need for prospective randomized-controlled trials to further test this hypothesis. Hoyles et al. designed a randomized controlled trial, the United Kingdom trial, in which 45 patients were randomized to receive either low dose prednisolone and 6 intravenous infusions of cyclophosphamide followed by oral azathioprine or to receive placebo. The trial was negative both for its primary endpoint, decline of forced vital capacity (FVC) and diffusing capacity for carbon monoxide (DLCO) at 12 months, as well as for its secondary end points, change of appearance in HRCT or dyspnea scores. There was, however, a trend toward statistical significance in the treatment arm of the trial, suggesting there likely was benefit of immunosuppressant therapy in SSc-ILD (51).

Further key evidence for the role of cyclophosphamide in the treatment of SSc-ILD came from the Scleroderma Lung Study-I (SLS-I) trial, the first positive trial in interstitial lung disease. The SLS-I trial was a 13-center double-blind, randomized, placebo-controlled trial in which 158 patients with symptomatic active alveolitis and SSc-ILD were randomized to receive either oral cyclophosphamide or placebo for 12 months. The patients were followed up for 12 more months after the end of the treatment period. The trial achieved its primary end point, demonstrating a statistically significant mean absolute difference in FVC at 12 months between the cyclophosphamide and the placebo arm of 2.53% in favor of the cyclophosphamide arm of the trial. In addition, the patients treated with cyclophosphamide had improvements in patient-centered outcomes including physiological improvements such as improved dyspnea, functional ability, health-related quality of life and reduced skin thickening (45). However, further subgroup analysis of the SLS-I trial patients showed that these improvements were not maintained at 24 months, except for a sustained improvement in dyspnea (60). This would suggest that ongoing immunosuppression is required in order to maintain the benefits of cyclophosphamide, but this needs to be balanced with the side effects of cyclophosphamide such as the risk of developing malignancy, infertility or hemorrhagic cystitis.

Interestingly, in the SLS-I trial there was a correlation between the extent of baseline fibrosis and the response to cyclophosphamide (45), which implies that patients with more active disease are a subgroup that would benefit more from treatment with cyclophosphamide. Given the significant side-effects associated with cyclophosphamide, it is important to balance the risk and the benefit of starting this medication.

Precisely the concern over cyclophosphamide-associated side effects prompted the design of the Scleroderma Lung Study 2 (SLS-II) trial, which compared oral cyclophosphamide against mycophenolate mofetil (MMF), an inosine monophosphate dehydrogenase inhibitor, which ultimately impairs lymphocyte proliferation and lymphocyte migration, for the treatment of SSc-ILD. A total of 142 patients with SSc-ILD that met specific criteria of breathlessness, lung function and HRCT findings were randomized to receive either MMF for 24 months or cyclophosphamide for 12 months followed by placebo for 12 months. The SLS-II trial failed to meet its primary end-point of change in forced vital capacity as a percent of the predicted normal values at 24 months. However, importantly it was noted that there was a higher drop-off rate from the cyclophosphamide arm of the trial and the time to stopping treatment was significantly shorter for the patients treated with cyclophosphamide. Side effects of leukopenia and thrombocytopenia were observed more frequently in the patients who received cyclophosphamide. However, a post-hoc analysis of the primary endpoint showed that there was substantial improvement in the adjusted FVC % from baseline to 24 months in both arms of the trial (46). Notably, SLS-II is the first trial demonstrating the effect of MMF in the treatment of SSc-ILD and suggested that the choice of therapy could be based on consideration of the side-effect profile.

The next development in the treatment options for SSc-ILD came from the SENSCIS (Safety and Efficacy of Nintedanib in Systemic Sclerosis) trial in which 576 patients with SSc-ILD with fibrosis affecting at least 10% of their lungs on HRCT were assigned to either receive the tyrosine kinase inhibitor nintedanib or placebo. The trial reached its primary end point with patients’ annual rate of FVC decline being −52.4 mls in the nintedanib group versus −93.3 mls in the placebo group. Given that systemic sclerosis affects younger patients, this is a significant cumulative effect of reduced rate of FVC decline over time. Subgroup analysis showed there was no difference in the effect of nintedanib depending on the extent of underlying fibrosis, suggesting that patients could benefit from nintedanib irrespective of their degree of underlying fibrosis. While nintedanib was shown to reduce the rate of decline of FVC, it had no effect on extra-pulmonary manifestations of systemic sclerosis such as skin changes measured by the Rodnan skin score. The most frequently encountered side effects in the nintedanib group were gastrointestinal, including diarrhea and were generally tolerable to patients (47). The SENSCIS trial was continued as an open-label extension, the SENSCIS-ON trial (A Trial to Evaluate the Safety of Long Term Treatment With Nintedanib in Patients With Scleroderma Related Lung Fibrosis, NCT03313180) in order to study the long-term effects of nintedanib. Data from the SENSCIS-ON trial has shown that the effect of nintedanib on reducing the rate of FVC decline is maintained beyond 52 weeks (61).

Nintedanib’s promise in the treatment of a broader range of non-IPF types of lung fibrosis was explored further by the landmark Efficacy and Safety of Nintedanib in Patients with Progressive Fibrosing Interstitial Lung Disease (INBUILD) trial. The design of the trial was based on the premise that there is a common pathological mechanism in different types of pulmonary fibrosis that can be targeted irrespective of the underlying cause of the fibrosing lung disease. In this phase 3, double-blind, randomized, placebo-controlled trial, a total of 663 patients with pulmonary fibrosis that involved at least 10% of the lungs on HRCT and was progressing despite treatment over the past 24 months and were recruited. The patients were also selected according to pulmonary function test criteria of FVC at least 45% predicted and diffusing capacity for carbon monoxide (DLCO) in the range between 30 and 80 percent predicted. The trial reached its primary end point, demonstrating a significantly lower annual rate of decline of FVC in the patients treated with nintedanib: −80.8 ml per year for the treatment arm compared to – 187.8 ml per year for the placebo arm (*p* < 0.001). Furthermore, patients with a UIP-like fibrotic pattern were another primary population within the study and demonstrated an even more pronounced difference of −82.9 ml per in the treatment arm versus – 211.1 ml in the placebo arm (*p* < 0.001) (48).

A subgroup analysis of patients with autoimmune-related ILD from the INBUILD trial was conducted that demonstrated among 170 subjects with autoimmune disease-related ILDs, the rate of decline in FVC over 52 weeks was −75.9 ml/year with nintedanib versus −178.6 ml/year with placebo (62). The findings of the INBUILD trial raise an important point regarding whether a splitting or a lumping approach toward diagnosing ILD should be adopted, given that regardless of the cause in cases of progressive fibrosing ILD, the anti-fibrotic agent nintedanib can slow the rate of decline of FVC.

Further clinical trials were designed based on knowledge of the pathophysiology of systemic sclerosis. Increased levels of the cytokine interleukin-6 (IL-6) have been reported in patients with systemic sclerosis, particularly the cohort of patients that exhibit cutaneous involvement early (63, 64). It has also been identified as one of the markers of progression in patients with milder versions of SSc-ILD (FVC > 70%) as the serum level of IL-6 is correlated with the progression of SSc-ILD (65) as well as with increased decline in FVC and increased mortality (66). The central role of IL-6 in the pathogenesis of systemic sclerosis is hypothesized to be in driving the initial immune-mediated inflammation toward profibrotic processes (67, 68). Tocilizumab is an anti-IL-6 receptor antibody which has already been approved for the treatment of several autoimmune diseases including rheumatoid arthritis, juvenile idiopathic arthritis and giant cell arteritis (69). Initial promising results for tocilizumab in the treatment of systemic sclerosis were obtained from the phase 2 trial faSScinate (A Study of RoActemra/Actemra (Tocilizumab) Versus Placebo in Patients With Systemic Sclerosis) in which patients with progressive systemic sclerosis randomized to tocilizumab showed an improvement in skin thickness and a reduced rate of decline of FVC; however, both of these changes were not statistically significant, indicating the need for a larger-scale randomized controlled trial (52).

The potential role of tocilizumab in treating patients with SSc-ILD was explored in the focuSSced (A Study of the Efficacy and Safety of Tocilizumab in Participants with Systemic Sclerosis) trial, a phase 3 multicenter, randomized, double-blind, placebo-controlled trial in which 210 patients with diffuse cutaneous systemic sclerosis for 60 months or less and a modified Rodnan skin score (mRSS) of 10–35 at screening were randomized to receive either a weekly injection of tocilizumab or to placebo for 48 weeks. Importantly the patients in the trial were stratified according to their IL-6 levels. It is notable that the patient group in the focuSSced trial were highly selected; these were patients that have early disease, given the inclusion criteria was patients who exhibited their first non-Raynaud symptom no earlier than 60 months prior to recruitment in the trial. Furthermore, they had cutaneous involvement, which is known to be associated with more rapidly progressive ILD and their disease was assessed to be active based on at least one marker of inflammation among CRP, platelets and ESR. An exclusion criterion was the use of other background immunomodulatory therapies (50).

The focuSSced trial failed to meet its primary endpoint of difference in change from baseline to week 48 in mRSS. However, a key secondary end-point of change in FVC from baseline to week 48 was met. Patients in the tocilizumab arm of the trial maintained their FVC at 48 weeks, while the patients receiving placebo showed a significant decline in their FVC. Importantly, this outcome was demonstrated both in the overall patient population and in the approximately two thirds of patients that had SSc-ILD – patients with SSc-ILD in the placebo arm demonstrated a reduction of FVC by 257 mls, while patients with SSc-ILD in the tocilizumab arm demonstrated a reduction of only 20 mls. The observed adverse effects were in line with the known side effects of tocilizumab including infections and cardiac events (50). The focuSSced trial suggests that tocilizumab can prevent the decline of FVC in selected patients with scleroderma whose disease is at a high risk of progression. A continuation of the focuSSced trial as an open-label study explored the effects of tocilizumab at 96 weeks and demonstrated that the slowing of decline in FVC was preserved and the long-term side effects were in line with the known side effects of tocilizumab (70).

Another aspect of the pathogenesis of SSc-ILD was targeted in the STRATUS (Systemic Sclerosis Abituzumab Study) trial (53). The STRATUS trial studied the effect of abituzumab, an antibody against αVβ6 integrin (71, 72), which is involved with the activation of TGFβ (73), in patients with SSc-ILD. In this Phase II study the aim was to enroll 175 patients with SSc-ILD on stable MMF dose that would be randomized to either receive intravenous abituzumab every 4 weeks for 104 weeks or placebo and the primary end point was the annual rate of change in FVC. The trial was, however, terminated early due to low enrolment – 24 patients in total were enrolled, which made it impossible to draw meaningful conclusions from the data due to the small sample size (53).

Given the important role of humoral immunity in the development of CTD-ILD as signified by the association of certain antibodies with the development of ILD, it was hypothesized that rituximab, an anti-CD20 antibody that depletes B-cells (74), would have a beneficial effect for the treatment of CTD-ILD. A 2018 study by Sircar et al. included 60 patients with dcSSc who were randomized to receive either rituximab or cyclophosphamide. After 6 months patients in the rituximab arm of the trial had a statistically significant improvement in FVC while those in the cyclophosphamide arm of the trial showed a decline in FVC. Furthermore, there were more adverse events observed in the patients taking cyclophosphamide (54). In the RECITAL (Rituximab versus cyclophosphamide for the treatment of connective tissue disease-associated interstitial lung disease) trial 101 patients with CTD-ILD were randomized to either receive cyclophosphamide or rituximab. The primary end-point was the rate of change of FVC at 24 weeks, which improved in both arms of the trial. There was a slightly greater improvement of 38 mls in FVC in the cyclophosphamide arm of the trial by 24 weeks, but this did not reach statistical significance (*p* = 0.493). The observed improvement in FVC was again maintained at 48 weeks in both arms of the trial, but without statistically significant difference (49).

In summary, both immunomodulatory and antifibrotic therapies play an important role in the treatment of CTD-ILD, with immunomodulatory therapies likely being more effective in earlier inflammatory stages of the disease and antifibrotic therapies in later fibrotic stages. There is significant potential for combining immunomodulatory and antifibrotic therapies, which is explored by ongoing trials (55).

## 5. Discussion

### 5.1. When treatment should be initiated

The management challenge in SSc-ILD stems from the highly variable course of the condition, which ranges from subclinical to life-threatening (75). The treatment dilemma arises from the fact that there is a subset of patients whose disease may be relatively stable and who could develop medication related side effects without associated benefits. On the other hand, early initiation of treatment in the appropriate clinical setting could halt the progression of fibrosis in other patients. The SSc-ILD is most likely to progress during the first 4 years of systemic disease and especially in the first 2 years and in a small subset of patients, in whom lung disease precedes the cutaneous manifestations of SSc^3^. The presence of a mild to moderate ILD early in the course of the systemic disease or the evidence of recent ILD progression from the clinical, lung function and imaging point of view, should lower the threshold for treatment initiation. A factor that plays an important role in the introduction of treatment is disease severity (76). A staging system has been devised (United Kingdom Raynauds and Scleroderma Association Staging System, UKRSA) that uses HRCT and FVC data to stratify the severity of disease and has been validated for prognostication (77). When ILD extent was obviously less than, or obviously more than, 20% of the total lung volume on rapid assessment, ILD can be defined as “mild” or “extensive,” respectively. In cases with an “indeterminate” disease extent (i.e., expert HRCT scoring would be required to classify disease), the FVC threshold of 70% can be used to define the ILD as mild or extensive. The distinction between mild and extensive lung disease was shown to be strongly predictive of mortality and subsequent disease progression. Treatment decisions can only be undertaken on a case-by-case basis, taking into account the views of the patient especially in mild forms of ILD. In case of mild disease in which often the introduction of treatment is not required, a strategy of close monitoring with repeat lung function tests every 4 months for at least 2 years could be applied to assess the behavior of ILD. An alternative approach highlighted by the focuSSced trial is early initiation of treatment in patients with mild forms of SSc-ILD who are deemed at high risk of disease progression based on a combination of demographic factors, lung function tests, radiological data and serum markers. Clinically reliable biomarkers to predict disease progression are still being developed and this is a promising approach for the future once these biomarkers become more reliable (78). On the other hand, in extensive disease treatment introduction is warranted to prevent progression of ILD. In any case, discussion in a dedicated multidisciplinary team (MDT) meeting should take place to decide the initial management approach (either observation or treatment), the change of medication in case of treatment failure and the most appropriate non-pharmacological approach.

### 5.2. Future directions

More refined understanding of the pathogenesis of CTD-ILD has aided the design of further clinical trials. Future possibilities for the treatment of CTD-ILD arise from elucidating the role Th2-driven immune response plays in the pathogenesis of systemic sclerosis. Romilkimab, an anti-IL-4 and anti-IL-13 antibody (79, 80), has shown promising results in animal models and is currently undergoing Phase II trials (Effectiveness and Safety of SAR156597 in Treating Diffuse Systemic Sclerosis, NCT02921971). Aside from using immunomodulatory therapies on their own, future directions for combined antifibrotic and immunomodulatory therapies in CTD-ILD will hopefully come from the Scleroderma Lung Study-3 (SLS-3) trial, which will compare the effect of the combination of pirfenidone and mycophenolate with mycophenolate and placebo on FVC at 18 months^55^. There is already promising data for pirfenidone from a recent randomized control trial conducted by Wang et al. who demonstrated that patients with SSc-ILD who receive both pirfenidone and immunosuppressants have a statistically significant improvement in FVC at 24 weeks compared to patients receiving only immunosuppressants (56). Furthermore, the LOTUSS study in which patients with SSc-ILD received pirfenidone for either 2 weeks or for 4 weeks demonstrated that pirfenidone has an acceptable side effect and safety profile, so pirfenidone is a promising antifibrotic therapy to continue exploring for patients with CTD-ILD (81). Another method of combining antifibrotic and anti-inflammatory therapies may be derived from the use of Janus kinase inhibitors (JAKi), which have both antifibrotic and anti-inflammatory actions and are currently being investigated for their potential role in the treatment of SSc-ILD (82).

### 5.3. Conclusion

CTD-ILD is a heterogenous group of conditions with a variable clinical course, which leads to challenges in their diagnosis and subsequent management. The ILD component of CTD is a major determinant of morbidity and mortality in CTDs and new developments from clinical trials have started yielding results to fill the urgent need for new therapies for CTD-ILD. The therapies for CTD-ILD remain an area of active research and hopefully there will soon be an even greater array of targeted therapies to choose from. Clinicians are still faced with the challenge of carefully selecting the most appropriate patients whose disease is likely to progress to start therapy on and balancing the risks and benefits of commencing therapy at the most appropriate time.

## Author contributions

All authors listed have made a substantial, direct, and intellectual contribution to the work and approved it for publication.

## Conflict of interest

The authors declare that the research was conducted in the absence of any commercial or financial relationships that could be construed as a potential conflict of interest.

## Publisher’s note

All claims expressed in this article are solely those of the authors and do not necessarily represent those of their affiliated organizations, or those of the publisher, the editors and the reviewers. Any product that may be evaluated in this article, or claim that may be made by its manufacturer, is not guaranteed or endorsed by the publisher.
